# A Novel Multishaped Variable Stiffness Magnetic Catheter for Minimally Invasive Surgery

**DOI:** 10.34133/cbsystems.0675

**Published:** 2026-09-01

**Authors:** Xingfeng Xu, Xinling Li, Siyang Zuo

**Affiliations:** Key Laboratory of Mechanism Theory and Equipment Design of Ministry of Education, Tianjin University, Tianjin 300350, China.

## Abstract

Magnetically controlled continuum robots enable dexterous manipulation within compact dimensions. However, their deformations are typically limited to C-shaped configurations, which restricts their practical applications such as obstacle avoidance, stable tip orientation, and navigation through anatomical channels with minimal tissue trauma. Moreover, the fixed stiffness of these robots limit its adaptability. To address these limitations, a continuum catheter must possess both multishaped deformation capability and variable stiffness. In this work, we propose a magnetically steerable catheter incorporating 2 radially magnetized magnets and a thermosetting shape memory polymer. Two variable stiffness segments that achieve a 20.8-fold stiffness variation (from 30 to 70 °C) within a compact design through shape memory polymer actuation. The distal magnet can rotate axially, while a single uniform magnetic field generates differential torques on both magnets, enabling multishaped deformation, including C-, J-, and S-shaped configurations. Large C-shaped deformation is achieved under a 15-mT uniform magnetic field, with a maximum distal bending angle of 127°. Experimental validation on the highly realistic gastrointestinal phantoms and ex vivo stomach model demonstrates the potential for clinical application. Compared with existing magnetic catheters, the proposed design exhibits enhanced deformation diversity and operational flexibility, making it adaptable to a broader range of medical procedures.

## Introduction

Minimally invasive surgery (MIS) has emerged as one of the most effective alternatives to conventional surgical procedures due to its advantages of smaller incisions, faster recovery times, and reduced operative risks [1–3]. Miniaturized medical instruments used in MIS are capable of reaching deep surgical sites through anatomical lumens or blood vessels, facilitating diagnostic and therapeutic procedures without the necessity of open wounds [4,5]. Among them, flexible continuum catheters exhibit superior flexibility and compliance compared to conventional tools. As a result, they effectively reduce the risk of infection, shorten procedure duration, and enable safer diagnostic protocols [6–10].

Despite these advantages, current flexible continuum catheters face several technical challenges, particularly the design conflict between miniaturization and dexterity. Various actuation mechanisms, such as tendon/cable-driven [11–15] and multibackbone motorized configurations [16–18], have been implemented in medical catheters to enhance dexterity. These mechanisms achieve multiple degrees of freedom for shape adaptation but typically require internal mechanical components such as micromotors or cable pulleys that impede miniaturization. Furthermore, transmission mechanisms in these actuation methods introduce frictional losses and hysteresis phenomena that compromise control precision.

Magnetic actuation has consequently gained marked research attention [19]. By embedding magnetic components within catheter structures, these devices enable controlled bending configurations through external magnetic fields [20–23]. Magnetic actuation eliminates the complex transmission systems, thereby facilitating both enhanced flexibility and reduced size simultaneously. However, current magnetic catheters exhibit limited C-shaped deformation patterns where tip orientation remains intrinsically coupled with catheter posture. To address this constraint, multiple variable stiffness (VS) segments achieve diverse bending configurations through selective shape locking [24]. Nevertheless, these designs necessitate rigid sections that pose safety risks during intraluminal navigation. In addition, shape transitions between configurations often exhibit temporal delays and sequential dependencies—for instance, the C/J-shaped deformation is the intermediate state of the S-shaped deformation.

MIS catheters must maintain sufficient flexibility for safe navigation through anatomical lumens while achieving adequate rigidity for stable positional maintenance and distal tool operation. Current VS solutions primarily use jamming techniques [25–27] and phase change materials [28,29]. Granular/particle jamming relies on negative pressure-induced interparticle friction for stiffness modulation but faces miniaturization limitations below 5 mm due to particle size constraints. Low-melting-point alloys (LMPAs) are a prevalent type of phase change material that provides substantial stiffness variation via solid–liquid transitions. Catheters based on LMPAs with small diameters can dispense with pneumatic channels and granular components [30,31]. However, potential toxic leakage necessitates rigorous encapsulation requirements that either reduce phase change material volume or increase overall dimensions. In contrast, thermosetting shape memory polymers (SMPs) demonstrate glass-to-rubber transition properties that provide substantial stiffness variation without molten-phase risks [32,33].

**Fig. 1.**
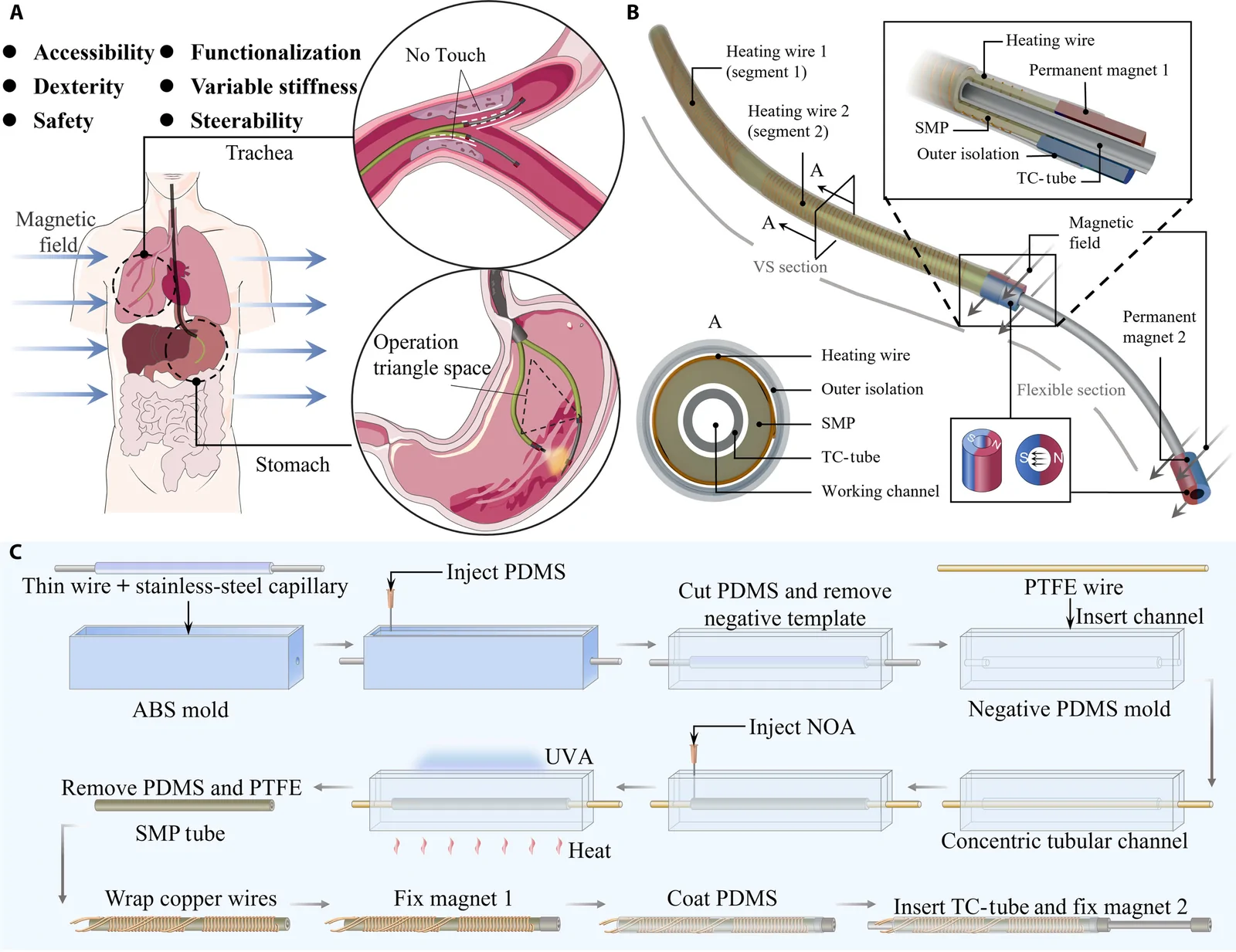
Shape memory polymer (SMP)-based variable stiffness (VS) magnetic catheter. (A) Clinical application potential. (B) Schematic illustration catheter design. (C) Fabrication process. UVA, ultraviolet A; ABS, acrylonitrile butadiene styrene; NOA, norland optical adhesive.

From the earlier considerations, we developed an SMP-based VS magnetic catheter, as illustrated in Fig. 1. The main novelty and contribution of this work are as follows: (a) We design a novel VS magnetic catheter that incorporates 2 heating elements to modulate the stiffness of the flexible shaft and 2 spatially separated radially magnetized magnets to achieve multishaped deformation; (b) with 2 independent VS segments and 2 spatially separated, radially magnetized permanent magnets, this catheter achieves further enriched deformation patterns, permitting complex C-/S-/J-shaped deformations with expanded workspace coverage; and (c) our proposed design substantially enhances flexibility and functionality while maintaining miniaturization, markedly expanding the application spectrum of robotic catheterization in MIS procedures.

## Materials and Methods

### Design and fabrication

In the present work, a biocompatible polyurethane SMP with dual thermal/ultraviolet curing capabilities is used, whose transition temperature varies with geometric configuration and curing parameters. Two independent enameled copper coils serve as heating elements to achieve segmented stiffness variation. Precise stiffness modulation is attained through controlled regulation of electric current parameters (amplitude and duration) and enables simultaneous compliance for accessible navigation and rigidity for operational stability.

The catheter features a VS section composed of 2 independent VS segments, along with 2 spatially separated, radially magnetized permanent magnets. Multiple VS segments further enrich deformation patterns, permitting complex C-/S-/J-shaped configurations with expanded workspace coverage. In addition, the mutually canceling magnetic responses of the 2 radially magnetized magnet configurations in specific states enable standby-mode operation unaffected by external fields, allowing simultaneous control of multiple catheters within an identical magnetic actuation system.

As shown in Fig. 1B, we propose a novel catheter that incorporates 2 spatially separated, radially magnetized magnets. Permanent magnet 2 is mounted on a torque coil tube (TC-tube) that enables remote rotation of the distal magnet and thus allows precise modulation of the relative magnetization angle between 2 magnets. These 2 magnets exhibit distinct responses in the uniform magnetic field. By modulating the relative magnetization directions between magnets, our design achieves complex C-/S-/J-shaped configurations with expanded workspace coverage. Notably, this design enables multishape deformations in flexible states without requiring complex magnetic field configurations or stiffness-dependent shape locking.

The catheter fabrication process, as illustrated in Fig. 1C, uses an injection molding approach. First, a 3-dimensional (3D)-printed mold containing a symmetrical center hole serves as a container, and a stainless-steel capillary serves as a negative template. Degassed polydimethylsiloxane (PDMS; Sylgard 184, Dow Corning) is then poured into this mold to form a transparent elastomeric matrix. The stainless-steel capillary is removed from the matrix, and a polytetrafluoroethylene (PTFE) mandrel (Ø0.6 mm) is inserted into the channel. Second, NOA86H resin is then injected into the transparent mold via precision syringe, and after curing, it forms an SMP tube (Ø1.9 mm in outer diameter, Ø1.2 mm in inner diameter, and 60 mm in length). Two independent enameled copper wires (Ø0.08 mm) wind around the SMP tube. A neodymium annular magnet (Ø2.2 mm in outer diameter and Ø1.2 mm in inner diameter) is bonded to the SMP distal end. Finally, PDMS encapsulation is applied through dip coating and thermal curing to establish the final insulation layer. The TC-tube (Ø1.0 mm in outer diameter and Ø0.6 mm in inner diameter) with premounted proximal magnet is finally assembled with the SMP component to complete the catheter fabrication.

The magnetic actuation system for applying externally driven fields is constructed using triaxial square Helmholtz coils. Three orthogonally arranged coil pairs generate uniform axial magnetic fields along the *x*, *y*, and *z* directions. Through superposition of these tridirectional magnetic fields, a 15-mT magnetic field is produced with arbitrary orientation in a cubic workspace (20 cm × 20 cm × 20 cm). The magnetic flux density generated by the coils exhibits proportional dependence on coil current and inverse proportionality to coil radius. By precisely controlling the current magnitude and direction in each coil pair, we achieve real-time regulation of both magnetic field intensity and orientation, thereby governing the catheter’s bending motions.

Complementing the magnetic actuation system, a driven package (see Fig. S1) controls global catheter advancement/retraction and assists catheter manipulation within the magnetic field. This driven package comprises 2 linear translation modules and 1 rotational module. The base translation module controls global catheter advancement/retraction, while the upper translation module regulates TC-tube movement to adjust the distance between the 2 magnets. The distal magnet’s rotation is achieved through a rotational module connected to the TC-tube. All modules are powered by DC motors and positioned externally to the magnetic actuation system. The catheter connects to the driven package through a high-density polyethylene tube.

### Deformation model

**Fig. 2.**
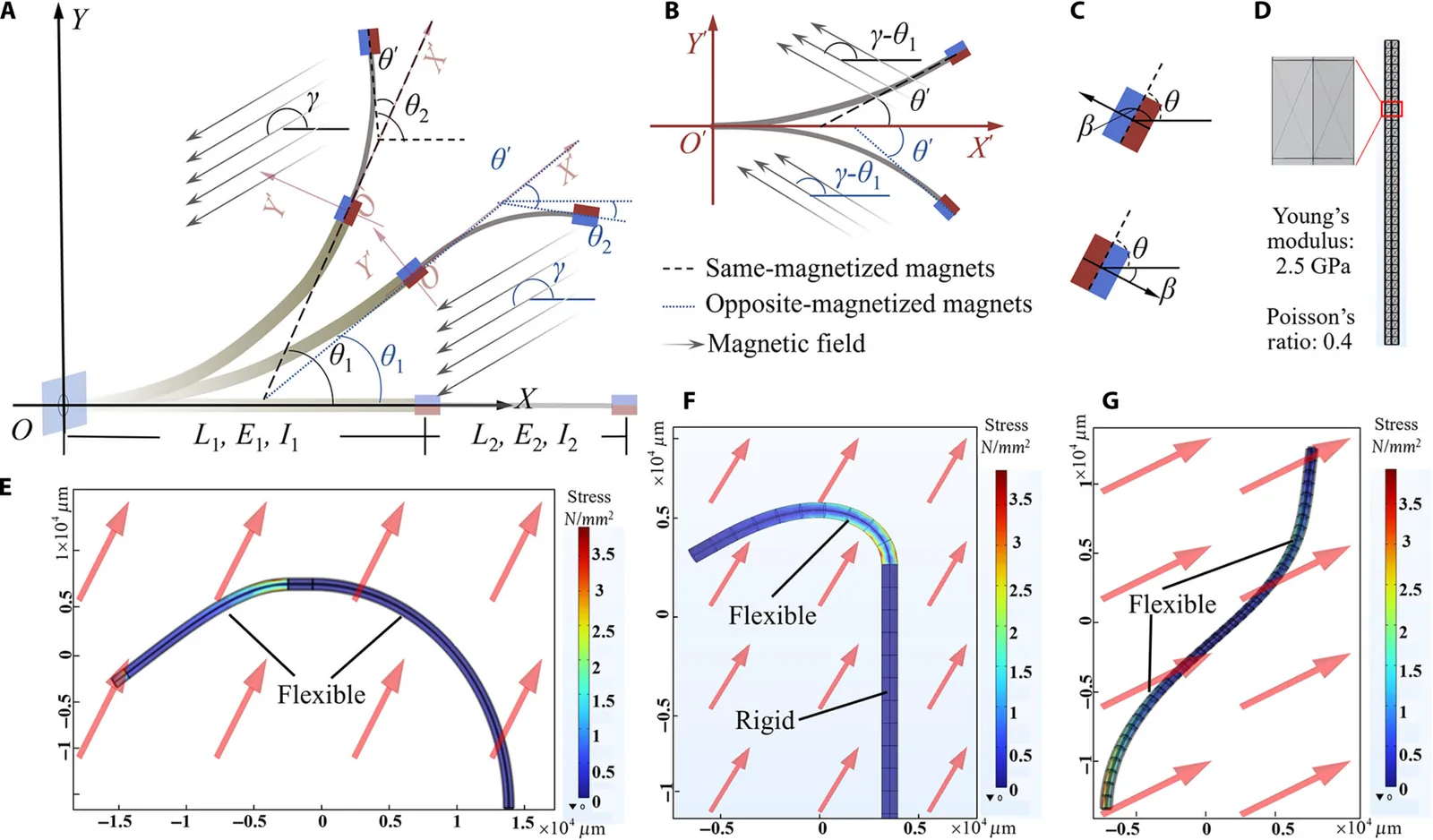
Bending model and simulation validation of the variable stiffness (VS) magnetic catheter. (A) Original coordinate system. (B) Transform coordinate system. (C) The relationship between axial angle and dipole moment angle. (D) Simulation setup. (E) Simulation results of C-shaped deformation. (F) Simulation results of J-shaped deformation. (G) Simulation results of S-shaped deformation.

For radially magnetized magnets, a 90° angular offset exists between the dipole moment orientation and the catheter’s distal direction, as illustrated in Fig. 2. The magnetic torque that a magnet experiences in a magnetic field is as shown in Eq. (1) [29].

$$T=T_{m}=\mathrm{mB}\;\text{sin}\left(\gamma -\beta \right)$$ (1)

Consequently, in a uniform magnetic field, the relationship between the deflection angle (*θ*) at the distal end of a catheter containing a single radially magnetized permanent magnet and the external field direction (*γ*) is given by Eq. (2).

$$\theta =-\frac{\mathrm{mBL}\;\text{cos}\left(\gamma -\theta \right)}{{\mathrm{EI}}_{d}}$$ (2)

where *L* is the catheter length, *m* is the magnetization intensity of the magnet, *β* is the dipole moment orientation angle, and *B* denotes the magnetic flux density of the uniform field. Our dual-magnet catheter design uses the coordinate system shown in Fig. 2A.

For the VS section, the total magnetic torque (*T*) equals the sum of torques applied to both magnets. The deflection angle (*θ*_1_) of the proximal magnet is determined by Eq. (4).

$$T=T_{1}+T_{2}=-\mathrm{mB}\;\text{cos}\left(\gamma -\theta_{1}\right)\mp \mathrm{mB}\;\text{cos}\left(\gamma -\theta_{2}\right)$$ (3)

$$\theta_{1}=-\frac{{\mathrm{mBL}}_{1}\left[\text{cos}\left(\gamma -\theta_{1}\right)\pm \text{cos}\left(\gamma -\theta_{2}\right)\right]}{E_{1}I_{1}}$$ (4)

where *L*_1_, *E*_1_, *I*_1_, and *θ*_2_ represent the length, elastic modulus, moment of inertia of the VS section, and deflection angle of the permanent magnet 2, respectively. The positive sign applies when the 2 magnets share identical magnetization directions, while the negative sign is used for opposing orientations. To establish the relationship between the distal magnet’s deflection angle (*θ*_2_) and the uniform field direction, we simplify the problem through coordinate transformation (Fig. 2C). Assuming that *θ*′ denotes the deflection angle in the new coordinate system, *θ*_2_ in the original system becomes:

$$\theta_{2}=\theta_{1}+\theta^{\prime }$$ (5)

$$\theta^{\prime }=\mp \frac{{\mathrm{mBL}}_{2}\;\text{cos}\left(\gamma -\theta_{2}\right)}{E_{2}I_{2}}$$ (6)

The negative sign in Eq. (6) is selected when the dipole moments of the distal and proximal magnets are initially aligned, whereas the positive sign applies when their dipole moments are initially opposed.

Finite element simulations were conducted in COMSOL to generate 3 distinct deformation shapes under different magnetic field configurations. The boundary conditions and mesh discretization of the model are illustrated in Fig. 2D. For each deformation mode (C-, J-, and S-shaped), specific magnetic field magnitudes and orientations were prescribed. In addition, the flexible state of the VS segment and the applied magnetic field strength are explicitly indicated in each simulation result in Fig. 2E to G. As shown, the catheter successfully achieved the 3 target configurations under the corresponding magnetic loading conditions.

### Experimental design

To systematically evaluate the proposed VS catheter, the experimental design is structured to validate its fundamental mechanical and thermomechanical functionalities.

#### Validation of multishaped deformation

The catheter was secured in a 3D-printed fixture and positioned at the center of a triaxial Helmholtz coil system. A high-resolution camera, aligned parallel to the catheter’s longitudinal axis, provided fixed-view imaging for positional tracking. With aligned dipole moments between the distal and proximal magnets under a 15-mT uniform field, both magnets experienced codirectional magnetic torques, forcing the catheter to bend toward alignment with the external field direction and generating a large-angle C-shaped deformation. Beyond C-shaped deformation, S-shaped deformation was realized by opposing the dipole moments of the 2 magnets while orienting the external field. Under opposed magnetization and a specific field orientation, the distal magnet experienced a greater torque magnitude than the proximal counterpart. This torque imbalance created asymmetric curvature distribution, resulting in a J-shaped deformation.

#### Validation of bending characteristics

**Fig. 3.**
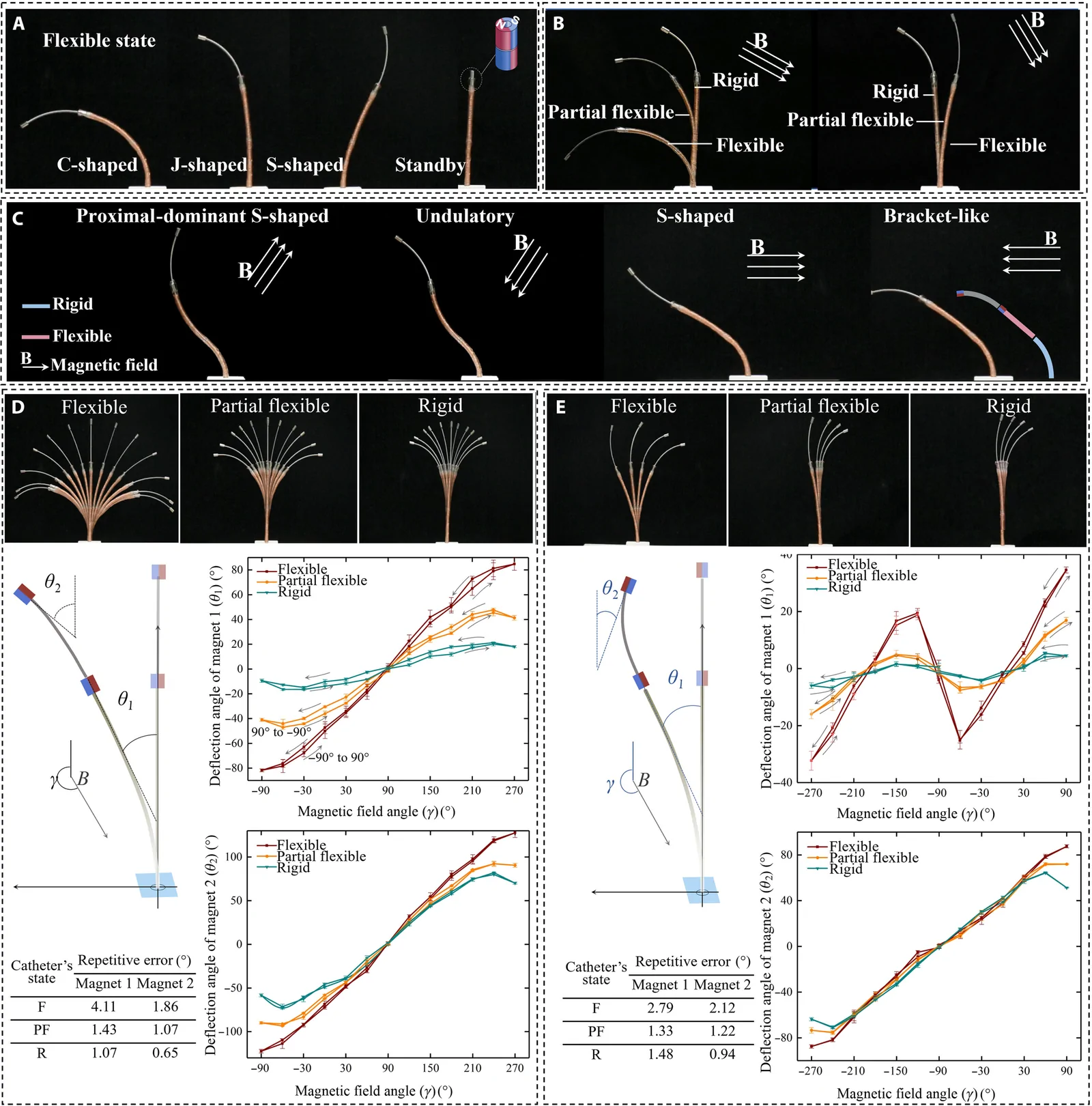
Deformation versatility of the magnetic catheter. (A) Multishaped deformation in flexible state, including C-, J-, and S-shaped configurations and standby mode. (B) Deformation responses under different stiffness states. (C) Representative programmable deformation modes of the catheter under variable stiffness and magnetic actuation, including proximal-dominant S-shaped, undulatory, S-shaped, and bracket-like deformations. (D) Bending characterization of same-magnetized magnets: schematic of magnet orientation variation, angle definition, and field-angle versus deflection-angle relationships. (E) Bending characterization of opposite-magnetized magnets: schematic of magnet orientation variation, angle definition, and field-angle versus deflection-angle relationships.

A high-resolution digital camera aligned parallel to the catheter was used to measure deflection angles of both magnets. The vertically upward direction shown in Fig. 3D was defined as 0°, with counterclockwise rotation designated as positive (+) and clockwise rotation as negative (−). Magnetic torque applied to the magnets reaches its maximum when the external field is perpendicular to the dipole moment, while minimal torque occurs when the dipole moment aligns with the magnetic field direction.

To investigate the catheter’s bending behavior and hysteresis under varying magnetic field orientations, the distal magnet’s dipole moment was first aligned with the proximal magnet. The external magnetic field angle was cyclically modulated as follows: starting from 90° (aligned with the dipole moment), rotating counterclockwise to 270°, returning to 90°, then sweeping clockwise to −90°, and finally reverting to 90°, completing one full cycle. Deflection angles of the catheter were recorded throughout the angular variations. Three complete cycles were tested for each of the 3 catheter states (flexible [F], partially flexible [PF], and rigid [R]), plotting the relationship curve between the deflection angle of the catheter and the magnetic field angle.

Subsequent tests reversed the distal magnet’s dipole moment relative to the proximal magnet, with the field cycled from −90° to 90° and back to −90°, followed by a sweep to −270° and return to −90°. Three cycles were similarly tested for each state.

#### Validation of thermomechanical characterization

**Fig. 4.**
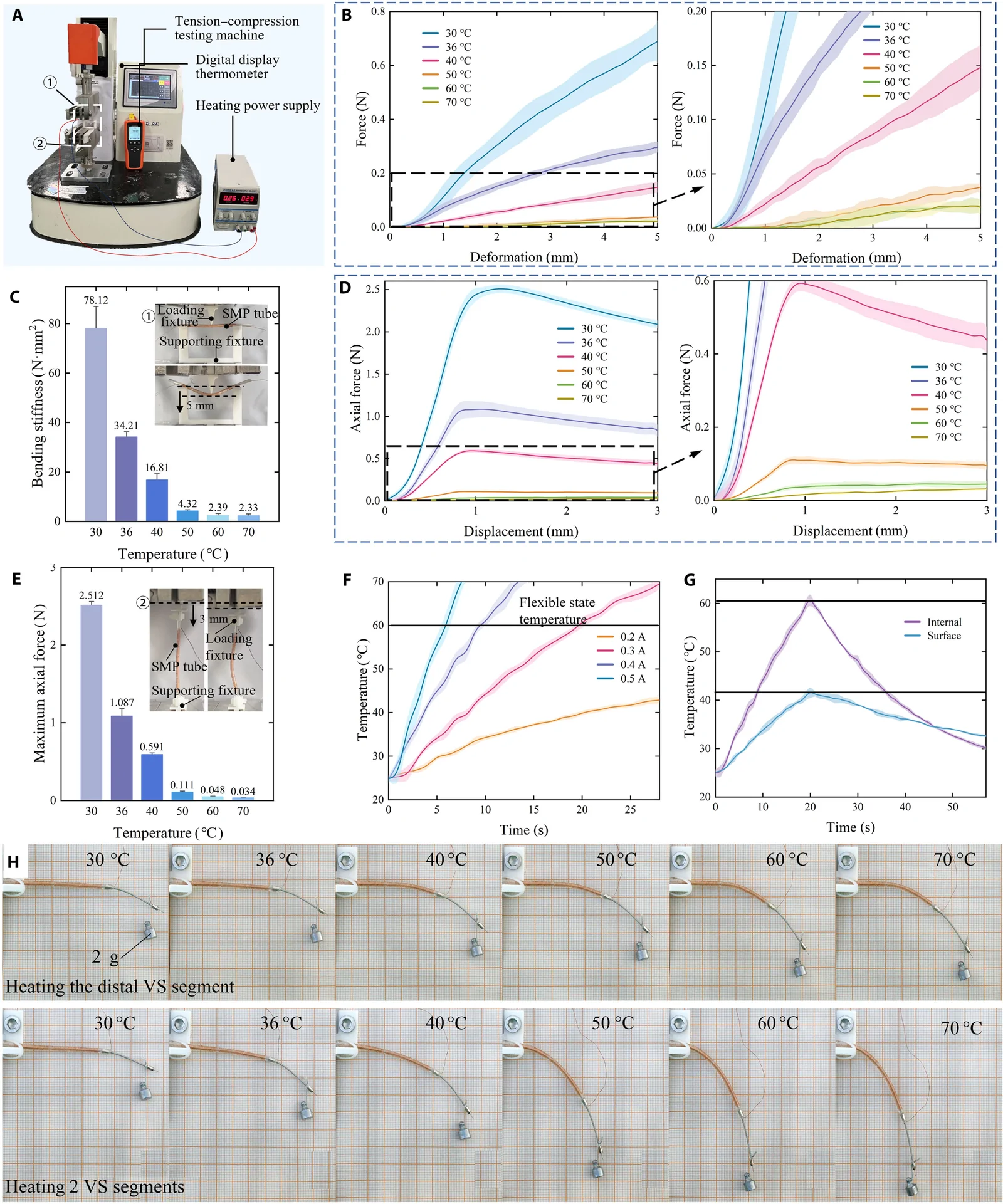
Thermomechanical and variable stiffness (VS) characterization. (A) Experimental setup for 3-point bending test and uniaxial compression test. (B) Force and deflection relationship curve. (C) Bending stiffness values at different temperatures. (D) Displacement and axial force relationship curve. (E) Maximum axial force at different temperatures. (F) Heating response under different applied currents. (G) Temperature evolution during heating and cooling at 0.3 A. (H) Load response of a cantilever beam under single and dual VS segment heating. (H) Load response of a cantilever beam under single and dual VS segment heating.

A heating system composed of enameled copper wire was wrapped around the sample, and the SMP tube temperature was monitored using a K-type thermocouple. Since surface temperature is a key safety criterion for the catheter, surface temperature measurements were conducted on the fully assembled device. A K-type thermocouple (Ø 0.1 mm in diameter) was placed on the outer surface of the insulation layer, and temperature data were collected using a high-precision thermometer. A current of 0.3 A was initially applied to heat the SMP tube from room temperature (25 °C) to target temperatures of 30, 36 (human body temperature), 40, 50, 60, and 70 °C. Upon reaching each target temperature, the current was reduced to 0.09, 0.13, 0.16, 0.20, and 0.23 A, respectively, to maintain thermal stability and prolong testing duration. The deflection and reaction force of the SMP tube were measured using a tension–compression testing machine, with custom 3D-printed support fixtures ensuring experimental consistency. The experimental setup is illustrated in Fig. 4A. The bending stiffness of the SMP tube at different temperatures was calculated using the following equation:

$$K=\mathrm{EI}=\frac{\partial P}{\partial S}\frac{L^{3}}{48}$$ (7)

where *P* is the reaction force, *L* is the span length, and *S* is the measured deflection of the tube.

## Results

This section is organized into 2 parts. The Fundamental mechanical and thermomechanical validation section presents the fundamental mechanical and thermomechanical validation experimental results corresponding directly to the experimental design described in the Experimental design section. The System-level functional validation section further provides system-level functional evaluations, including dexterous manipulation capability, and simulated clinical feasibility demonstrations.

### Fundamental mechanical and thermomechanical validation

#### Multishaped deformation

Our design introduces a standby mode absent in conventional magnetic catheters. When the flexible segment retracts to its mechanical limit under opposing magnets, near-equal counteracting torques cancel magnetically induced deformation (Fig. 3A), maintaining stability in compliant states and enabling controlled positional retention without bending.

The VS section further diversifies deformation shapes through 3 stiffness states: flexible (F), partially flexible (PF), and rigid (R), as illustrated in Fig. 3B. By strategically modulating stiffness distribution along the VS section, both curvature and distal orientation can be actively regulated (Fig. 3C). With both VS segments flexible in a C-shaped configuration, rigidifying the second subsegment and reversing distal magnetization produce a proximal-dominant S-shaped deformation. Cooling the first subsegment to rigidity while adjusting the external field induces undulatory deformation. Varying distal magnetization and field angle generates an S-shaped, while a J-shaped deformation in first subsegment with a rigid second subsegment yields a bracket-like profile with high distal curvature. These programmable state transitions demonstrate the system’s capability to adaptively reconfigure curvature profiles for diverse clinical scenarios. Overall, the proposed catheter enables on-demand shape reconfiguration and controllable state transitions to satisfy varying procedural requirements.

#### Bending characterization

To investigate the catheter’s bending characteristics, we measured the deflection angles of both magnets as shown in Fig. 3D. Under a 15-mT magnetic field, the distal end achieved maximum bending angles of 127° (flexible state), 93° (partial flexible state), and 80° (rigid state), while proximal magnet deflections measured 85°, 47°, and 21°, respectively. These results confirm that the catheter achieves large-angle bending in the flexible state while effectively suppressing curvature in the rigid state under identical field intensity.

As shown in Fig. 3E, S-shaped bending occurred between −90° and 0°, with optimal S-configuration observed at −60°, characterized by opposing deflections of the proximal and distal magnets. J-shaped deformation dominated between 0° and 90°, where both magnets deflected codirectionally, but the flexible distal segment exhibited markedly higher curvature than the VS section. Curvature responses symmetrical from −90° to 90° were observed during field sweeps from −90° to −270°. In the flexible state, the catheter achieved high-curvature S-shaped deformation and instantaneous S-to-J shape transitions without hysteresis. The rigid state enhanced the mechanical constraint on the VS section, amplifying the angular disparity between the 2 magnets to produce J-shaped profiles with intensified distal curvature.

Across all states, the catheter demonstrated high repeatability (maximum angular error: 3.9°, in flexible state) and low hysteresis. Furthermore, isotropic bending performance was observed, with consistent angular responses regardless of field sweep direction.

#### Thermomechanical characterization

To characterize the relationship between the stiffness variation of the VS section and temperature, as well as to determine the operational temperature range, we performed 3-point bending tests on SMP tubular samples with identical inner and outer diameters to the SMP layer of the designed VS segment.

To prevent mechanical failure of the SMP tube during rigid-state measurements, we constrained the maximum allowable deflection to 5 mm. Following 5 repeated trials at each temperature, the mean value curves were obtained after data processing, which are presented in Fig. 4B.

The stiffness of the SMP tube at 6 temperatures is presented in Fig. 4C. The bending stiffness change factor (SCF) between 30 °C and other temperatures (human body temperature 36, 40, 50, 60, and 70 °C) was calculated as 2.28, 4.65, 18.08, 32.67, and 33.53, respectively. However, the integration of the TC-tube in the fully assembled catheter reduced the effective SCF values. With the TC-tube exhibiting a bending stiffness of 1.5 N·mm^2^, the practical SCF of the catheter between 30 °C and other temperatures was measured as 2.22, 4.34, 13.68, 20.67, and 20.78, respectively.

Uniaxial compression tests were subsequently conducted on the SMP tube to further investigate its thermomechanical properties. The tests were performed using a compression testing machine at a constant crosshead speed of 40 mm/min, compressing the SMP tube by 3 mm axially to measure the maximum axial force it could withstand at different temperatures. Temperature monitoring and heating protocols were identical to those used in the 3-point bending tests.

The collected displacement and load data were plotted as curves, as shown in Fig. 4D. The SMP tube exhibited linear stress–strain behavior prior to buckling, followed by bending deformation after reaching critical compressive force, after which no further increase in axial resistance was observed. The critical compressive forces measured at 30, 36, 40, 50, 60, and 70 °C were 2.51, 1.08, 0.59, 0.11, 0.05, and 0.03 N, respectively (Fig. 4E). This progressive reduction in critical force with increasing temperature quantitatively demonstrates the SMP’s thermally activated stiffness modulation capability.

Heating time and cooling time constitute critical parameters for evaluating the rigidity–flexibility transition efficiency, necessitating systematic characterization. To investigate the influence of applied current on heating rates and quantify heating duration, we measured temperatures of the SMP tube that was heated under varying currents (0.2, 0.3, 0.4, and 0.5 A) through the enameled copper wire. Prior to each measurement, the SMP tube was cooled to ambient temperature (25 °C). Five repeated trials were conducted for each current level, with heating curves processed and plotted, as shown in Fig. 4F. The SMP tube could be heated from 25 to 60 °C in 20, 10, and 6 s under 0.3-, 0.4-, and 0.5-A currents, respectively. At 0.2 A, the SMP tube temperature rose to 47 °C and subsequently exhibited negligible variation; therefore, heating time was not calculated for this condition.

To characterize cooling time, we heated the SMP tube to 60 °C and subsequently allowed it to cool naturally in ambient air. The cooling time, defined as the duration required for the temperature to decrease from the flexible state (60 °C) to 30 °C, was measured as 36 s.

For the standalone SMP tube, applying 0.3-A current for 20 s resulted in a temperature of 60 °C. Under identical heating conditions (0.3-A current for 20 s), the surface temperature of the assembled catheter reached approximately 41 °C, demonstrating safe operation within the biocompatible temperature range (Fig. 4G). The cooling time for the catheter surface temperature, measured from 41 to 30 °C under natural convection, was 54 s.

In practical operation, the SMP tube works in an intermittent heating mode. A brief heating pulse (a few seconds) softens the SMP tube for shape reconfiguration. Once the target configuration is reached, the heating phase is then terminated, allowing the SMP to quickly return to its rigid supporting state. Hence, sustained heating is unnecessary, and the transient thermal response shown in Fig. 4G faithfully represents the actual operating condition, with the surface temperature staying around 41 °C.

To directly evaluate the segmented VS performance and practical bending characteristics of the magnetically controlled catheter robot, we conducted a single-arm cantilever load test. The proximal end of the catheter was fixed to a 3D-printed fixture, while a vertically positioned backplane with grid paper served as a reference background. A high-resolution camera, aligned parallel to the backplane, captured the catheter’s bending deformation and tip displacement under various states. A 2-g weight was suspended from the catheter’s distal end, and bending displacements were observed while modulating the temperature of the VS segments, as shown in Fig. 4H.

Initially, only segment 2 was heated to a specific temperature, and the resulting deformations and displacements were recorded. Visually, the distal displacement and curvature of the 60-mm bendable section increased progressively with rising temperature. Furthermore, as shown in Fig. 4H, the unheated segment 1 maintained structural stability throughout, confirming the catheter’s effective segmented stiffness modulation.

After resetting the catheter to its initial state, the entire VS section was heated uniformly. Under the same loading conditions, a longer bendable length (resulting from uniform heating of the whole VS section) produced greater distal displacements. This observation, also included in Fig. 4H, validates the system’s load-bearing adaptability across different bendable lengths.

### System-level functional validation

#### Dexterity

**Fig. 5.**
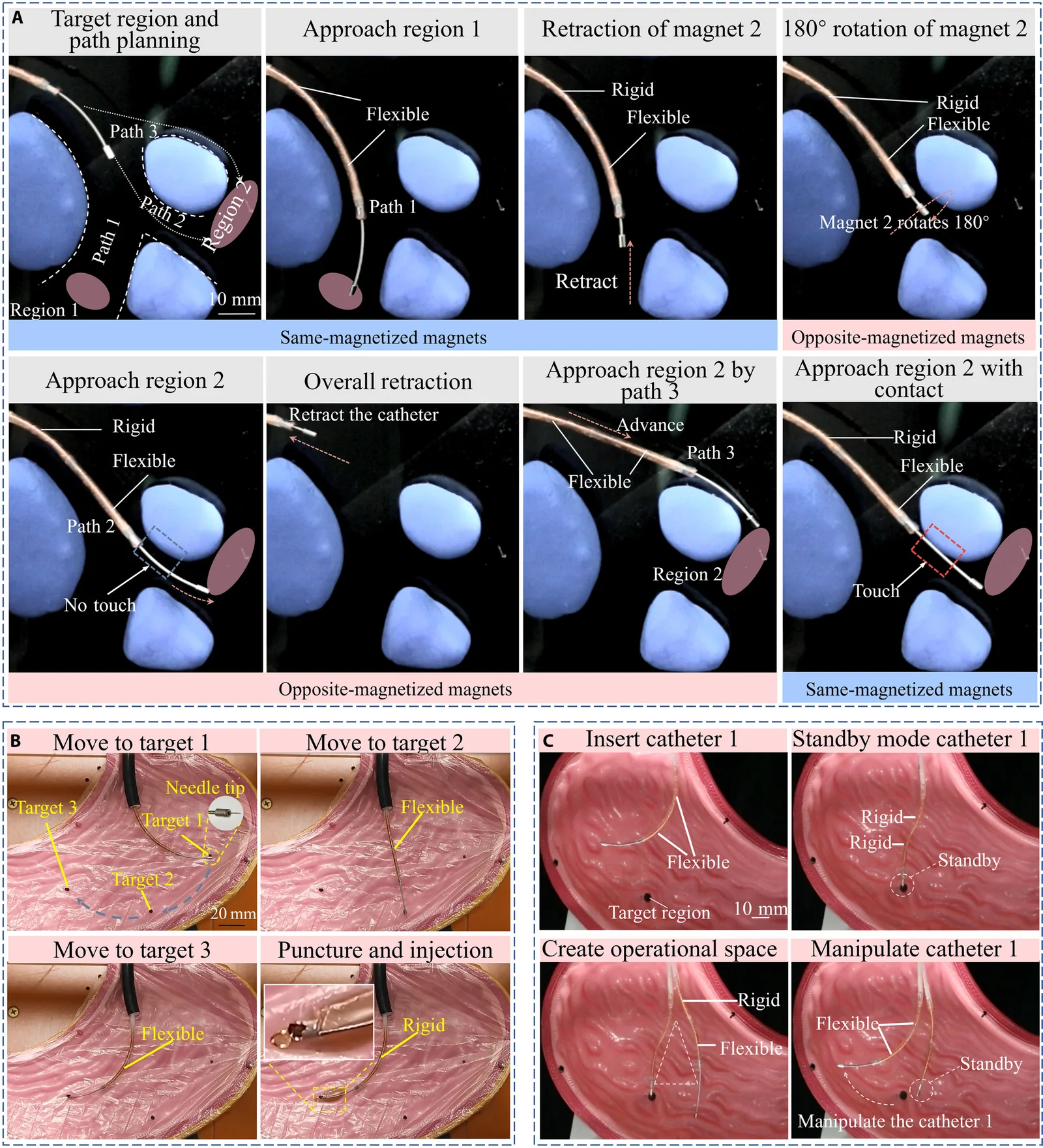
Functional validation of the magnetic catheter. (A) Obstacle avoidance demonstration. (B) Submucosal injection demonstration. (C) Multicatheter cooperative operation demonstration.

With multishaped deformation capabilities, catheters can enhance their flexibility and path selection ability, allowing them to adapt to diverse anatomical paths. We conducted an obstacle avoidance verification experiment to test its capability to bypass obstacles and navigate through branched paths. The catheter was guided through a channel with specifically arranged obstacles and bifurcations, successfully circumventing obstacles to reach target regions (Fig. 5A).

Typically, with the dipole moments of the 2 magnets aligned, the linear translation module of the auxiliary drive system advances the catheter from the entry point. Under the influence of an external magnetic field, the catheter bends in a C-shape. This deformation provides uniform curvature and enables large-angle deflection at the distal end, facilitating overall turning to bypass obstacles and traverse bifurcations to reach region 1.

Subsequently, by reversing the dipole moment of the distal magnet and applying a magnetic field at a specific angle, the catheter adopts an S-shaped deformation. This configuration allows navigation through winding bifurcations to reach region 2 without external channel constraints. During the adjustment of the distal magnet’s dipole moment, the upper linear translation module retracts the flexible segment to avoid contact with obstacles. Conventional catheters lacking diverse deformation inevitably collide with obstacles or channel walls when navigating through S-shaped bifurcations. Such contact may cause tissue friction, potentially leading to vasoconstriction, vascular injury, and complications. The multishaped deformation capability of the catheter is expected to reduce the friction-related damage during MIS.

In addition, by adjusting the direction of the external magnetic field, the catheter can perform J-shaped deformation to navigate path 2 and reach region 2. In scenarios where certain paths are impassable (e.g., due to obstructions or the need for multidevice coordination), the catheter’s multishaped deformation capability increases the likelihood of selecting alternative paths to reach target regions (Movie S1).

#### Phantom experiments

The integration of functional components at the catheter’s distal end (such as injection needles, graspers, or complementary metal-oxide semiconductor cameras) demonstrates the system’s expanded capabilities and clinical applicability. The VS design enables flexible navigation in the compliant state while providing rigid structural support after cooling for medical interventions. A perfluoroalkoxy Teflon capillary tube, connected to a 0.25-mm-outer-diameter needle tip, is threaded through the catheter’s working channel and secured at the distal assembly to create a functionalized device.

For validation, a plastic gastric model with synthetic membranes simulated submucosal injection procedures (Fig. 5B and Movie S2). The catheter navigates through endoscopic lumens to the stomach in its flexible state (F), guided by external magnetic fields to targeted positions. Sequential deployment allows distal tip positioning near observation points, transitioning to a rigid state (R) upon reaching lesions for membrane puncture. During needle penetration, the VS segment maintains structural integrity to counteract reaction forces. Concurrently, distal magnet rotation under magnetic torque optimizes needle tip orientation, facilitating controlled tissue penetration. Postpuncture confirmation, the syringe is depressed to administer saline or therapeutic agents, with flow control systems enabling precise dosage regulation in clinical applications.

Certain clinical applications require the simultaneous manipulation of multiple magnetic instruments within the workspace of an electromagnetic navigation system. For instance, in endoscopic procedures, a camera-equipped device must first locate and visualize the target lesion in real time, while a second instrument performs therapeutic interventions such as submucosal injection, puncture, or dissection. However, conventional magnetic catheters, which utilize permanently magnetized components, inherently experience continuous magnetic responses to external fields within the workspace. This persistent interaction prevents the undisturbed operation of additional magnetic tools, severely limiting multi-instrument workflows.

While existing VS catheters can mechanically lock into position by transitioning to rigid states, residual magnetic forces and torques inevitably induce tip oscillations. Furthermore, these systems cannot operate auxiliary instruments while maintaining flexible states, substantially restricting their utility. Our catheter design addresses these challenges through a standby mode that eliminates magnetic interference, enabling sequential control of multiple tools. Fig. 5C illustrates a clinical application scenario that requires stable operational characteristics.

This scenario (Movie S3 and Fig. S2) leverages the standby mode’s ability to neutralize magnetic interactions, enabling sequential tool activation while maintaining mechanical stability. By decoupling inactive instruments from the external field, the system overcomes the limitations of conventional designs, enhancing precision in multitool endoscopic interventions and expanding procedural versatility.

**Fig. 6.**
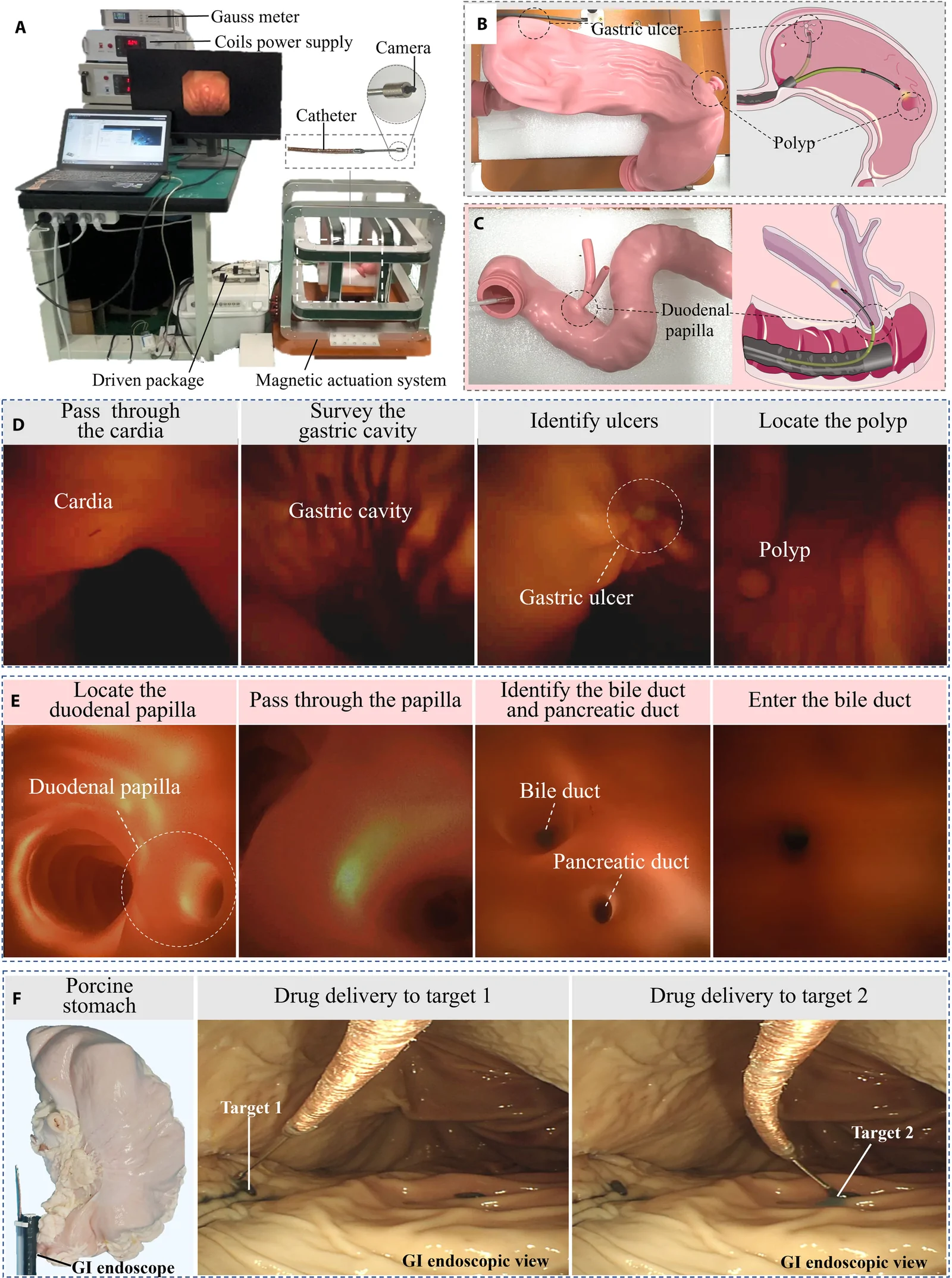
Experimental validation of the expanded capabilities and clinical applicability of the magnetic catheter. (A) Magnetic actuation system. (B) Gastric phantom for navigation evaluation. (C) Biliopancreatic duct phantom for endoscopic intervention. (D) Lesion detection in the gastric phantom. (E) Lesion detection in the biliopancreatic duct phantom. (F) Ex vivo porcine stomach experiment for targeted delivery validation. GI, gastrointestinal.

To further demonstrate the potential clinical applicability of the designed catheter, we conducted a model trial of lesion detection in the gastric and biliopancreatic duct, using an integrated camera to direct observation of ductal pathologies during interventions. The catheter was equipped with a miniature camera (optical density = 0.65 mm) fixed at its distal end. The camera cable was routed through the catheter’s working channel to an external processor, with real-time imaging displayed on a monitor. The experimental setup and anatomical models are shown in Fig. 6.

Under the camera guidance, the catheter navigated through the cardia into the relatively open gastric cavity (Fig. 6D). Magnetic field actuation enabled lesion location: A gastric fundus ulcer was located, followed by deflection to survey the gastric cavity, ultimately detecting a polyp at the gastroduodenal junction. This sequence validated the catheter’s diagnostic capabilities for gastric pathologies, highlighting its potential in endoscopic procedures. For biliopancreatic applications, the catheter replicated endoscopic retrograde cholangiopancreatography workflows: traversing the pylorus into the duodenum, magnetically steering to locate the duodenal papilla (Fig. 6E), and successfully entering the simulated biliopancreatic duct under visual feedback. Clinically, such catheters may serve as a submicroscope of a duodenoscope, enhancing the efficiency of biliopancreatic procedures through coordinated navigation. The catheter’s viability for visualized intraluminal intervention was experimentally confirmed (Movie S4). According to our statistical data, the magnetically controlled operation times for 2 phantom experiments—the submucosal injection procedure and the model trial of lesion detection in the gastric and biliopancreatic duct—were 76 and 38 s, respectively. Both were under 2 min, further confirming the clinical applicability of this catheter.

To further validate the translational potential of the proposed system, we conducted a targeted delivery experiment in an enclosed ex vivo porcine stomach (Fig. 6F and Movie S5), which provides a realistic cavity and surgical environment for evaluating the catheter’s performance. The catheter was inserted through an external working channel of a gastrointestinal endoscope. The endoscope was advanced through the cardia into the stomach, where the drug delivery procedure was performed under real-time endoscopic guidance. A miniature sprayer was integrated at the catheter tip and magnetically guided to access the target lesion site. The results demonstrated stable positioning and controllable delivery performance within the ex vivo tissue environment, without causing damage to the surrounding tissues. These findings provide preliminary evidence for the feasibility of the proposed catheter in minimally invasive gastric therapeutic procedures and highlight its potential for future clinical translation.

## Discussion and Conclusion

In this study, we designed and fabricated a novel flexible catheter with multishaped deformation capabilities and VS. The proposed catheter integrates 2 radially magnetized magnets, providing enhanced control over the proximal section and greater operational freedom compared to conventional single-magnet catheters. The actuation mechanism enables controlled rotation of the distal magnet, thereby allowing adjustment of the relative magnetization direction between the 2 magnetic elements. When the magnets are aligned, the catheter achieves large C-shaped deformations under a 15-mT uniform magnetic field, with a maximum distal bending angle of 127°. Opposing magnetization enables S- and J-shaped deformations under the same magnetic field, with symmetric curvature adjustments achievable through magnetic field direction modulation. Transition between deformation shapes requires no additional time, relying solely on external field adjustments or magnet reorientation. The VS functionality allows flexible navigation through complex lumens while providing rigid-state stability for interventions. The integration of multiple VS segments introduces additional degrees of freedom, enabling complex deformations. For example, the proximal section can remain rigid to avoid contact with delicate tissues, while the distal section retains flexible capabilities for lesion exploration.

Comparative analysis with existing studies in Table 1 highlights unique advantages: Our catheter achieves diverse deformation in flexible states, unlike prior designs requiring partial rigidity for mode switching or lacking segmented stiffness control [29,31–33]. While Mattmann et al. [33] use multiple VS segments and magnets, both their positions and orientations are fixed, limiting the achievable deformation flexibility. In our design, however, the 2 magnets allow adaptive adjustment of their relative position and magnetic field direction, which greatly enhances deformability control. Moreover, the mutually canceling magnetic responses of the 2 radially magnetized magnets in specific states enable a standby-mode operation unaffected by external fields, allowing simultaneous control of multiple catheters within an identical magnetic actuation system. In stiffness modulation, our design exhibits a 20.8-fold stiffness change from 30 to 70 °C, outperforming Piskarev et al. [32] (21-fold change but over a broader 22 to 80 °C range with only 2.4-fold change from 22 to 60 °C) and Lussi et al. [31] (faster transitions but smaller diameter and lower SCF).

**Table 1.** Comparison with other variable stiffness (VS) magnetic catheters

Description	Chautems et al. [29]	Lussi et al. [31]	Piskarev et al. [32]	Mattmann et al. [33]	This work
Outer diameter/mm	2.5	1	2	3	2.3
Materials	LMPA	LMPA	SMP	SMP	SMP
Number of deformable segments (VS + distal flexible part)	3 + 0	1 + 0	2 + 0	4 + 0	2 + 1
Number of permanent magnets/orientation	1/axially	1/axially	2/axially	4/axially	2/radially
Permanent magnet poses adjustable	No	No	No	No	Yes
Stiffness change factor (SCF)	–	5.5	21 (22–80 °C)	≈21 (30–80 °C)	20.8 (30–70 °C)
Transition time from rigid to flexible modes/s	15	10	27	9	10
Transition time from flexible to rigid modes/s	80	30	90	60	54

LMPA, low-melting-point alloy; SMP, shape memory polymer.

Structurally, the catheter’s elegant design facilitates manufacturing. A clearance between the torque tube and SMP layer prevents torsional instability during rotation, although this slightly compromises stiffness modulation efficiency. Potential improvements include smaller diameter torque tubes to promote miniaturization design, lubricant coatings to improve accessibility, and enhanced polytetrafluoroethylene wire alignment during fabrication to ensure consistent bending characteristics.

Thermomechanical testing confirmed highly reproducible temperature–stiffness relationships. SMP stiffness plateaus above 60 °C, suggesting operational limits below this threshold. At physiological temperatures (36 °C), the SMP exhibits a 14.33-fold stiffness change, while the integrated catheter achieves 9.3-fold. Uniaxial compression tests demonstrated that rigid-state SMP (30 to 36 °C) withstands significant axial loads, providing stable support, while flexible-state SMP (60 °C) minimizes tissue injury risks.

Stiffness transition efficiency relies on Joule heating (0.4 to 0.5 A optimal for controllability) and passive cooling. PDMS insulation limits surface temperatures to 41 °C during 60 °C SMP heating. For the VS section of this design, a temperature exceeding 60 °C is considered to achieve the ideal flexible state. According to prior studies on temperature-responsive biometamaterials for gastrointestinal applications [34], such short-term localized heating is safe in the digestive tract. The PDMS coating provides thermal insulation and convective heat transfer in liquid environments (e.g., stomach or blood vessels) and rapidly reduces the outer surface temperature, minimizing the risk of thermal injury to surrounding tissue.

The cooling time of 54 s for the VS section, from flexible to rigid, primarily serves to stabilize the catheter after it reaches the target location, rather than for continuous real-time switching. Considering that typical clinical procedures, such as gastrointestinal or bronchial interventions, last 30 to 120 min, this transition represents a relatively small portion of the overall procedure and is therefore within an acceptable range. Transition times could be further reduced through thermal design optimization or active cooling strategies to enhance responsiveness and operational efficiency. Although the internal TC-tube reduces the effective SCF from the intrinsic value of 20.8, the assembled device maintains sufficient stiffness variation for clinical scenarios such as gastrointestinal or bronchial interventions, where the catheter passes through natural lumens and thus does not encounter complex high-resistance tissues.

Motion precision testing revealed the catheter achieves peak bending angles at 150° field orientation in rigid/partially flexible states, attributed to torque reduction beyond 90° alignment. Opposed magnetization configurations showed constrained proximal deflection, addressable through magnet size or field intensity optimization. Repeatability errors (<3.9°) stem from field orientation inaccuracies, improvable via enhanced current control. Obstacle avoidance tests confirmed contact-free navigation with a fully rigid VS section, although minimizing rigid parts enhances in vivo safety.

A formal inverse mapping framework represents a natural extension for future work, enabling precise computation of magnetic inputs to achieve target tip positions, orientations, and curvature distributions. Existing methods [35,36] for soft robot motion planning and inverse mapping could be applied to further enhance systematic control. Incorporating such inverse mapping with closed-loop feedback would facilitate collision-free navigation and high-precision control in clinically relevant scenarios.

In conclusion, our catheter combines compact dimensions with superior flexibility, multishaped deformation capability in flexible states, and broad stiffness modulation (SCF 20.8). The catheter’s distal rotates axially, enabling differential torques under a uniform magnetic field for multishaped deformations (C-/J-/S-shaped). The J-shaped configuration facilitates endoscopic retrograde cholangiopancreatography by enabling sharp bending for common bile duct cannulation. The S-shaped configuration aids pulmonary bronchoscopic interventions by navigating double-curved airways (e.g., main bronchus to segmental bronchus). These examples highlight how the proposed design enhances procedural efficiency and reduces tissue trauma in real clinical applications. Compared to existing magnetic catheters, the proposed design offers greater deformation diversity and operational flexibility, making it suitable for a wider range of medical procedures. Experimental validations in gastric and biliopancreatic ducts underscore its clinical potential for intraluminal navigation and interventions. Future work will integrate active cooling and force sensing, along with time-resolved measurements, to further quantify switching dynamics and operational efficiency, thereby advancing performance and clinical applicability.

## Data Availability

Data sharing is not applicable to this article as no new data were created or analyzed in this study.
