# Supplementary material for: A Novel Multishaped Variable Stiffness Magnetic Catheter for Minimally Invasive Surgery
Source: Cyborg Bionic Syst. 2026 Sep 1;7:0675. doi: 10.34133/cbsystems.0675 (PMC13530375; doi:10.34133/cbsystems.0675)
Supplement: Supplementary file 1 [file cbsystems.0675.f1.zip › Supporting Information_revised.docx]

Supporting Information

**A Novel Multi-Shaped Variable-Stiffness Magnetic Catheters for Minimally Invasive Surgery**

*Xingfeng Xu, Xinling Li, Siyang Zuo**

**SUPPLEMENTARY MOVIES**

**Movie S1.** Obstacle avoidance for dexterity verification.

**Movie S2.** Submucosal injection based on simulation model.

**Movie S3.** Operating of multi magnetron catheter.

**Movie S4.** Model trial in gastric and biliopancreatic duct.

**Movie S5.** Ex vivo porcine stomach model experiment.

**SUPPLEMENTARY TEXT**

**Workflow for clinical application scenario that requires stable operational characteristics:**

Catheter 1 initially navigates in its flexible state, achieving large-angle bending under magnetic guidance (Fig. S2A). Segment 1 of the variable-stiffness catheter is cooled to achieve rigid state. The catheter is then manipulated to position its tip near the target location, establishing an S-shaped deformation (Fig. S2B). Subsequently, segment 2 is cooled to maintain this S-shaped deformation in standby mode, which facilitates the formation of a surgical operational triangular zone and enables subsequent insertion and magnetically-driven manipulation of a second catheter (Fig. S2C). Catheter 2 is then advanced, with its state and the external field angle adjusted to position its end near the target area, synergizing with Catheter 1 to maintain the triangulation zone (Fig. S2D, E, and F). After completing the intervention, Catheter 2 transitions to standby mode, reactivating Catheter 1 to enable it to receive magnetic response (Fig. S2G, H). Remarkably, even in a partially flexible (PF) state, Catheter 2 exhibits negligible magnetic interference due to the standby mode (Fig. S2I). Both catheters are ultimately retrieved without operational conflicts. Catheter 1 can be equipped with a CMOS camera at its distal end, while catheter 2 may carry therapeutic actuators at its terminal section.

**Figure S1.** Driven package.

**Figure S2**. Operating of multiple magnetron catheter under magnetic field: (A) insert catheter 1; (B) transform the deformation shape; (C) catheter 1 in standby mode; (D) insert catheter 2; (E) the proximal VS segment of catheter 2 becomes rigid and forms a surgical triangle with catheter 1; (F) manipulate catheter 2 to reach the target area; (G) catheter 2 in standby mode; (H) activate catheter 1; (I) manipulate catheter 1 in flexible state.

**Figure S3.** Conceptual illustration of a multi-shaped variable-stiffness magnetic catheter that integrates two radially magnetized magnets and a thermosetting shape memory polymer for minimally invasive surgery.
